# Molecular environment and atypical function: What do we know about enzymes associated with Mucopolysaccharidoses?

**DOI:** 10.1186/s13023-022-02211-1

**Published:** 2022-03-04

**Authors:** Weijing Kong, Cheng Lu, Yingxue Ding, Yan Meng

**Affiliations:** 1 Department of Pediatrics, Beijing Friendship Hospital, Capital Medical University, Beijing, 100050 China; 2 Department of Pediatrics, Chinese PLA General Hospital, Beijing, 100853 China; 3 Beijing Hong Jian Medical Device Company, Beijing, 100176 China

**Keywords:** Mucopolysaccharidoses, Molecular environment, Atypical function

## Abstract

Mucopolysaccharidoses are a group of lysosomal storage disorders caused by deficiency of enzymes involved in glycosaminoglycans degradation. Relationship between mucopolysaccharidoses and related enzymes has been clarified clearly. Based on such relationship, lots of therapies have been commercialized or are in the process of research and development. However, many potential treatments failed, because those treatments did not demonstrate expected efficacy or safety data. Molecular environment of enzyme, which is essential for their expression and activity, is fundamental for efficacy of therapy. In addition to enzyme activities, mucopolysaccharidoses-related enzymes have other atypical functions, such as regulation, which may cause side effects. This review tried to discuss molecular environment and atypical function of enzymes that are associated with mucopolysaccharidoses, which is very important for the efficacy and safety of potential therapies.

## Introduction

**Fig. 1:**
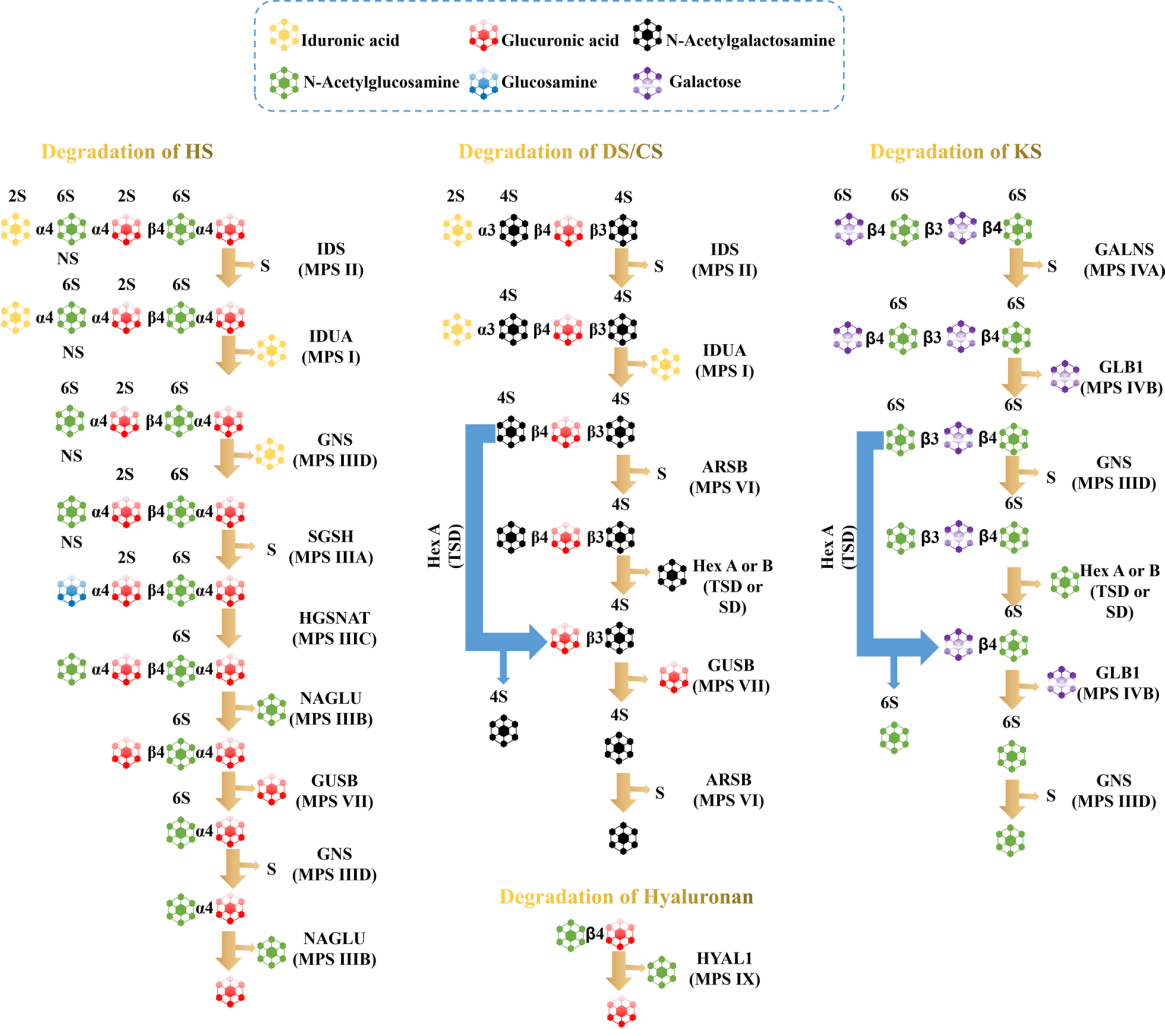
Degradation process of GAGs chains and enzyme malfunction in each step. Hex A or B: β-N-Acetylhexosaminidase A or B; TSD: Tay-Sachs disease; SD: Sandhoff disease; CS: chondroitin sulfate; DS: dermatan sulfate; HS: heparan sulfate; KS: keratan sulfate

Mucopolysaccharidoses (MPSs) are rare lysosomal storage disorders caused by abnormal accumulation of glycosaminoglycans (GAGs), which is due to deficiency of enzymes involved in degradation of GAGs. MPSs are categorized into seven subtypes. Six subtypes of MPSs (type I, III, IV, VI, VII and IX) are inherited in an autosomal recessive manner, while mucopolysaccharidosis (MPS) II is X-linked (1). There are eleven MPSs-related enzymes, including α-l-iduronidase (IDUA) for MPS I, iduronate sulfatase (IDS) for MPS II, heparan N-sulfatase (SGSH) for MPS IIIA, α-N-acetylglucosaminidase (NAGLU) for MPS IIIB, heparan acetyl CoA: α-glucosaminide N-acetyltransferase (HGSNAT) for MPS IIIC, N-acetylglucosamine-6-sulfatase (GNS) for MPS IIID, N-acetylgalactosamine-6-sulfatase (GALNS) for MPS IVA, β-galactosidase (GLB1) for MPS IVB, N-acetylgalactosamine-4-sulfatase (ARSB) for MPS VI, β-glucuronidase (GUSB) for MPS VII and hyaluronidase (HYAL1) for MPS IX (2) (Fig. 1).

Deficiency of MPSs-related enzymes causes abnormal accumulation of GAGs in lysosomes and subsequently induces clinical manifestations of MPSs, so reducing GAGs is the main purpose of treatment for MPSs. There are two specific ways to treat MPSs based on pathogenic mechanism: (1) recovery of enzyme activity, including enzyme replacement therapy (ERT), gene therapy (GT), and hematopoietic stem cell transplantation (HSCT); (2) reduction of GAGs accumulation in lysosome, such as substrate reduction therapy (SRT) (3, 4). At present, there are five treatments for MPSs that were approved by Food and Drug Administration (FDA), including laronidase for MPS I, idursulfase for MPS II, elosulfase alfa for MPS IVA, galsulfase for MPS VI and vestronidase alfa for MPS VII (Table 1) (5).All those approved treatments are tried to correct disease through recovery of enzyme activity.

**Table 1:** Approved therapies for MPSs

Name of syndrome	Deficient enzyme	Generic name	Brand name	Applicant	Approved date (FDA)
MPS I	IDUA	Laronidase	Aldurazyme^®^	BioMarin	April 30, 2003
MPS II	IDS	Idursulfase	Elaprase^®^	Shire	July 24, 2006
MPS IVA	GALNS	Elosulfase alfa	Vimizim^®^	BioMarin	February 14, 2014
MPS VI	ARSB	Galsulfase	Naglazyme^®^	BioMarin	May 31, 2005
MPS VII	GUSB	Vestronidase alfa	Mepsevii^®^	Ultragenyx pharm	November 15, 2017

Enzyme activity is the results of a series of complex processes, from genetic polymorphisms to protein degradation (6). Lots of factors in this complex process constitute the “molecular environment”. Molecular environment can affect MPSs-related enzymes at RNA level and protein level, and subsequently affect efficacy of therapies (7–9).

MPSs-related enzymes not only degrade GAGs but also have other atypical functions, such as inducing exocytotic process and promoting cell growth (10, 11). Except degradation of GAGs, all other functions of MPSs-related enzymes can be considered as “atypical functions” in this review. These atypical functions may cause side effects, which raise concerns about safety of therapies.

To our best knowledge, there was no review focused on molecular environment and atypical functions of MPSs-related enzymes, which are fundamental for efficacy and safety of therapies for MPSs. In this review, we tried to summarize molecular environment and atypical functions of enzymes associated with MPSs, although there are few related articles.

## Molecular environment

### RNA level (Expression)

#### Promoter function

Promoter is the region where certain regulatory elements bind to turn a gene on or off. If treatments are designed with original promoter of genes for MPSs-related enzymes, functions of these binding sites and regulatory elements should be considered. DNA sequencing and bioinformatics analysis can offer details of promoter. Promoter region of *IDS* gene was predicted to contain a large CpG island (CpG46) and 5 sites for transcription factor binding, whereas the 3’-untranslated region contains 5 microRNA (miRNA or miR) targeting sites. These elements may contribute to regulation of *IDS* gene in brain and other neural tissues (12). 5’-flanking region of GALNS lacks a canonical TATA box and CCAAT sequences, but it is GC rich (70.5%) and contains four GC boxes, one of which is supposed to be a binding site of regulatory element (13). Based on details of promoter, quantitative real-time PCR and other technologies can help researchers to find regulatory elements for MPSs-related genes. Fibroblast growth factor 2 (FGF-2) cannot induce expression of GALNS by itself, but expression of GALNS is induced by FGF-2 cooperating with runt-related transcription factor 2 (14). Estrogen response element binding sites, bound by estrogen receptors, were found in promoter of GALNS (15). However, different types of estrogen may cause different results: 17β-estradiol down-regulates expression of GALNS (15); estradiol benzoate increases activity of GALNS (16). By analyzing promoter of HYAL-1, Lokeshwar et al. (2008) found that this sequence had binding consensus sites for specificity protein 1(SP1), early growth response protein 1 (Egr1), activating enhancer-binding protein-2 (AP-2) and nuclear factor κB (NFκB), which was confirmed by chromatin immunoprecipitation assay (17). Meanwhile, two methylation sites, part of the SP1/Egr1-binding sites, epigenetically regulates HYAL-1 expression (17).

#### Bidirectional gene pairs

Bidirectional gene pairs are defined as two genes on different strands with adjacent 5’ ends (within a region of one kb) (18). Gene pairs are evolutionarily conserved and maintained for some functional reasons, including chromatin organization, DNA repair, and metabolism functions (19). *GALNS* and *TRAPPC2L* are classified as bidirectional gene pairs, because they are organized in head-to-head orientation with less than 1.0 kb between their transcript units and share a bidirectional promoter. This gene pair is modulated by 17β-estradiol via estrogen receptors in MCF-7 breast cancer cells (15). For therapies development in the future, gene pairs may inspire new treatments for MPSs.

#### miRNAs

In addition to those factors in genome, other elements should be monitored during treatment, because these molecules may affect transcription of genes associated with MPSs, including miRNAs, hormone and nutrient deprivation.

miRNAs are small endogenous RNAs that regulate various cellular and biological processes, such as inflammation and pyroptosis (20). *GNS* is the target of miR-675, miR-140 and miR-17 (21, 22). Up-regulation of miR-675 significantly down-regulates expression of *GNS* in K562 cells with silencing ferritin heavy chain (21). To face negative energy balance, miR-140 and miR-17 are up-regulated, which causes *GNS* to be down-regulated. In addition to *GNS*, *ARSB* is also directly regulated by miR-154-5p through interacting with 3’-untranslated regions of *ARSB* (23).

#### Alternative mRNA splicing

Alternative mRNA splicing, during which numerous messenger RNAs are generated and subsequently transcript encoding proteins of varied functions from the same gene, is an important mechanism to regulate protein function in different cells (24, 25). Alternative splicing is regulated by the interaction between cis-acting regulatory sequences and corresponding trans-acting regulatory proteins. For example, exonic splicing enhancers are recognized by serine/arginine-rich protein (8, 26). *IDS* gene generates three major different *IDS* transcripts (2.1, 5.4 and 5.7 kb), which are the result of alternative polyadenylation site selection. These different transcripts have the same open reading frame and encode the same protein (27). Unlike *IDS* gene, two alternatively spliced mRNAs are from *GLB1* gene, and encode two different proteins (28). Several mRNA splice variants of *HYAL1* were reported to have different activities in different cells. Five alternatively spliced variants of *HYAL1* encode enzymatically inactive proteins (29). Different splicing variants from *HYAL1* gene, with different functions, affect behavior of cells and are predictors of cancer (29, 30). Genes with several mRNA splice variants should be modified to improve efficacy of treatment, especially for HSCT and GT.

#### Peptide and hormone

Peptide and hormone also affect expression and activity of MPSs-related enzymes. LL-37, the only human cathelicidin-family host defense peptide, alone or in combination with IL17A concomitantly induces expression of *HYAL1* in human synovial sarcoma cell line SW982 (31). In fibroblasts, expression of *HYAL1* was induced by platelet-derived growth factor (PDGF)-BB through activation of extracellular signal-regulated kinase (ERK) mitogen-activated protein kinase (MAPK) and phosphoinositide 3-kinase (PI3K) pathways (32). Glucocorticoids and long-acting β2-agonists (LABAs) reduce expression and activity of *HYAL1* in airway smooth muscle cells (33, 34). As a cell senescence marker, *GLB1* is regulated by many factors. In LNCaP prostate cancer cells, expression of *GLB1* is increased after androgen deprivation treatment, which is the standard treatment for prostate cancer (35). In human endothelial cell line, tumor necrosis factor-alpha (TNF-α) induced expression of *GLB1*, meanwhile expression of *GLB1* is inhibited by allopurinol and apocynin (36). In idiopathic pulmonary fibrosis derived cells, re-expression of proliferator activates receptor gamma co-activator 1-alpha (PGC1-α) and modestly reduced expression of *GLB1* (37).

#### Nutrient deprivation

Another factor that affects expression and activity of enzymes associated with MPSs is nutrient deprivation, so nutritional status should be noticed during treatment. Expression of *SGSH* was increased in retinal pigment epithelial cell line-19 that had been subjected to nutrient deprivation for 48 h (38). In fibroblasts serum-starved for 7 days, activity of GLB1 is induced by many factors, such as regulatory associated protein of mTOR complex 1 (RPTOR) and RPTOR independent companion of mTOR complex 2 (RICTOR) (39).

### Protein level (activity)

#### Protein post-translational modifications

Protein post-translational modifications (PTMs) increase functional diversity of the proteins(40). Formylglycine is a catalytically essential residue that is found in the active sites of type I sulfatases (41). Sulfatase modifying factor 1 (SUMF1), a formylglycine-generating enzyme, can activate 17 known human sulfatases through transformation of conserved cysteine residue to c-alpha formylglycine (42). When cDNA of SUMF1 is co-delivered with a sulfatase cDNA via adeno-associated virus (AAV) vector and lentivirus (LV) vector to cells from MPSs patients, enhancing sulfatase activity can contribute to clearance of the intracellular GAGs (43). The results indicated that co-delivery of SUMF1 could enhance efficacy of GT in several sulfatase deficiencies (43). Results of a phase I/II trial of SAF301 (AAV vector serotype rh.10 carrying human SGSH and SUMF1 cDNAs) presented good safety with moderate improvements in behavior, attention and sleep disturbances (44).

Besides alteration of cysteine residue to c-alpha formylglycine, phosphorylation at C-6 of mannose residue is also a common PTM for sulfatases (45). Naz et al. (2013) used search tool for recurring instances of neighbouring genes (STRING) analysis and listed the top 20 proteins, including most members of UDP glucuronosyltransferase1 (UGT) family, which showed close interaction with GUSB (46). UDP-Nacetylglucosamine, with the help of enzyme UGT, phosphorylates mannose residue of GUSB. Phosphorylation at C-6 of mannose residue is recognized by mannose 6-phosphate receptor (M6PR), which is very important for translocation of GUSB from Golgi apparatus to lysosomes (47). Because phosphorylation of mannose residue can promote cellular delivery of lysosomal enzymes, lots of therapies are operated based on this mechanism. Chinese hamster ovary cells or human cell lines cannot generate recombinant human NAGLU (rhNAGLU) with mannose 6-phosphate during post-translational processing, so efficacy of rhNAGLU for MPS IIIB patients is limited by inadequate cellular delivery (48). Fusion with insulin-like growth factor 2 (IGF2) is an option to solve this problem, because IGF2/M6PR results in marked enzyme uptake in targeting tissue through enhanced lysosomal targeting (49). A phase 1/2, open-label study demonstrated that ICV-administered BMN 250 was well tolerated without treatment-emergent serious adverse events and presented good clinical effect (keeping total HS of CSF and liver volume in normal range; improvement in developmental quotient) (50).

If enzymes of ERT are not hoped to be delivered freely, avoiding M6PR interaction is a good choice. Modified SGSH is produced through chemical modification to recombinant human SGSH to partially disrupt glycan structures and preserve catalytic activity. Modified SGSH can reduce uptake of enzyme into peripheral tissues and facilitate distribution of modified SGSH in the central nervous system (51). After repeated intravenous administration, modified SGSH sustains higher concentration in serum, cerebrospinal fluid and brain interstitial fluid, which is in accordance with reduction of heparan sulfate and improvements of neuroinflammation (51).

#### Protein–protein interactions

Protein-protein interactions are physical contacts established between two or more proteins (52). Proteins operate localization or combine their substrate or achieve other objectives through “functional contact” (52). Egasyn, a non-specific carboxyl esterase, is an endoplasmic reticulum resident protein. Combination between egasyn and GUSB is essential for lysosomal targeting of GUSB, but neither the esterase active site of egasyn nor the C terminus of GUSB is involved in their interaction (46, 53). In the absence of CD44, HYAL1 cannot cleave hyaluronate in living cells (54). Assembling GLB1 with protective protein cathepsin A (PPCA) and neuraminidase 1 (NEU1) to form lysosomal multi-enzyme complex is essential for enzymatic activity of GLB1 (9). For the next step of ERT and GT development, these co-factors of MPSs-related enzymes should be noticed to achieve better efficacy.

#### Substrate analogues

Substrate analogues are similar in nature to substrates; however, substrate analogues are different from true substrates in their binding to the active site. Carrageenan is widely used to improve the texture and solubility of foods, including infant formula and nutritional supplements (55). Configuration of the 4-SO4 group of κ-carrageenan is similar to that of chondroitin 4-sulfate, so carrageenan mimics chondroitin-4-sulfate and dermatan sulfate to serve as the substrate of ARSB (56). Enzyme activity assays proved that κ-carrageenan could inhibit activity of ARSB and affect inflammation and insulin signaling (56, 57). These results indicated that patients with MPSs should pay attention to their diet and level of estrogen when they are being treated. Sulfatases, including IDS and ARSB, play important roles in metabolism of steroid hormones and of GAGs with the same active-site, so steroid hormones may affect activity for clearance of GAGs (58). In MCF7 cells and T47D cells, estradiol exposure was proved to inhibit activity of ARSB significantly (59). Following exposure for 6 days to different estrogen hormones, activity of GALNS, ARSB and IDS was reduced significantly in MCF7 cells (59). During treatment, these substrate analogues of GAGs should be monitored to get better results.

## Atypical function

Although MPSs-related enzymes demonstrate exquisite substrate specificity and little functional redundancy, these enzymes also have other atypical functions. Low delivery to target places and off-target effects cause high concentration of enzymes at unexpected places. Combination of high concentration and atypical functions of MPSs-related enzymes may cause adverse events which can lead to concern about safety of treatment options. Influence of reduction of enzymes is not included in this part, because all clinical manifestations of MPSs patients are caused by deficiency of enzymes.

Overexpression of IDS can activate exocytosis to induce enhanced glucose-induced insulin secretion in INS1E cells through phosphorylation of protein kinase C-alpha (PKC-α) and myristoylated alanine-rich C kinase substrate (MARCKS) (10). The mechanism under which IDS stimulates exocytosis remains unknown, however, this atypical function of IDS may explain some adverse events. From a clinical trial of ERT for MPS II, urticaria and skin rash, which were easily controlled with administration of antihistamines, may be induced by too many enzymes (60, 61).

Fusion gene of NAGLU and IKZF3 has tumourigenic effects in colorectal cancer (11). Compared to cells overexpressing only IKZF3, overexpression of NAGLU-IKZF3 significantly increased cell growth and migration, which hinted at a potential role of NAGLU in regulation of cell growth and migration (11).

**Fig. 2:**
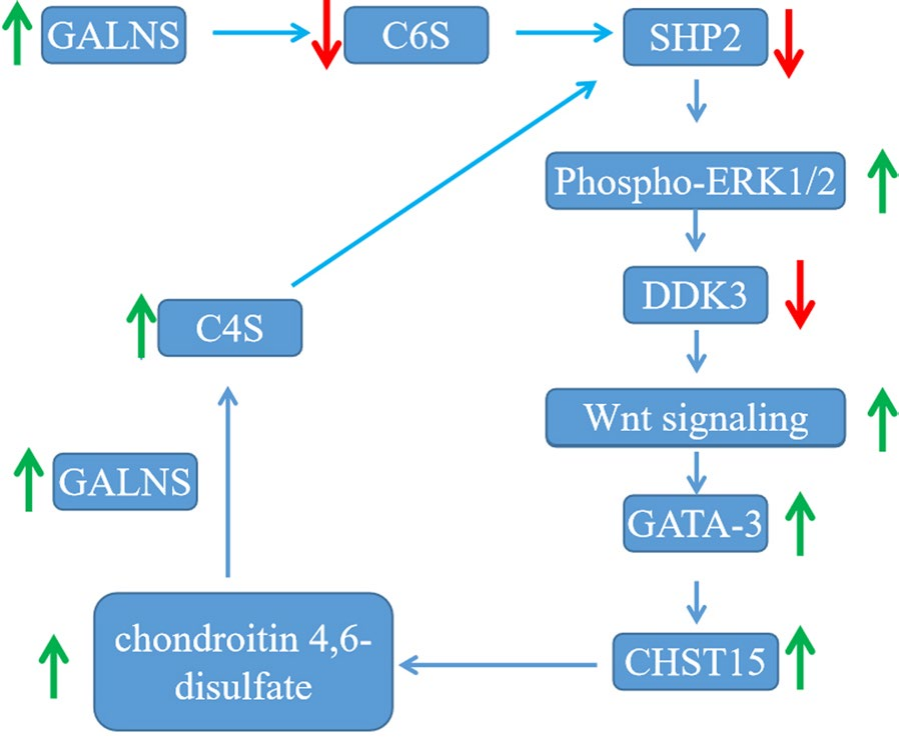
Overexpression of GALNS can induce Wnt signaling pathway. GALNS: N-acetylgalactosamine-6-sulfatase. C6S: chondroitin 6-sulfate. SHP2: Src homology 2 domain-containing phosphatase 2. ERK: extracellular signal-regulated kinase. DKK3: Dickkopf Wnt signaling pathway inhibitor. GATA-3: GATA Binding Protein 3. C4S: chondroitin 4-sulfate

In human prostate cells, overexpression of GALNS can induce Wnt signaling pathway by effects on Src homology 2 domain-containing phosphatase 2 (SHP2), phospho-ERK1/2, and Dickkopf Wnt signaling pathway inhibitor (DKK3) (62, 63). Overexpression of GALNS reduces the amount of chondroitin 6-sulfate, which causes chondroitin 4-sulfate to combine with more SHP2 and reduces activity of SHP2. Activity reduction of SHP2 activates phospho-ERK1/2, then DKK3 is suppressed, and Wnt signaling pathway is activated. Activation of Wnt signaling pathway increases the amount of carbohydrate sulfotransferase 15 (CHST15) to synthesize more chondroitin 4, 6-disulfate, which can be transformed to chondroitin 4-sulfate by GALNS. Increasement of chondroitin 4, 6-disulfate reduces the activity of SHP2 and re-activates Wnt signaling pathway (Fig. 2) (64). As a proto-oncogene, GALNS should be noticed during treatment for MPS IVA patients due to their impact on Wnt signaling pathway.

GLB1 deficiency causes MPS IVB and GM1-gangliosidosis. Therapies for MPS IVB are rarely reported, but GT, ERT and SRT have been explored to treat GM1-gangliosidosis, which can give some hints about atypical functions of GLB1. When GLB1 levels are augmented by GT or ERT for prolonged periods, GLB1 has the potential to promote secondary NEU1 deficiency (65, 66). An additional safety issue of GT for MPS IVB was mis-localization of GBL1 in the endoplasmic reticulum when GLB1 was over-expressed (9).

## Discussion

Knowledge of molecular environment and atypical functions of enzymes will be helpful for improving efficacy and safety of therapies for MPSs. To date, there has been no review about this topic. Our research aimed to draw more attention about molecular environment and atypical functions of enzymes associated with MPSs.

HSCT is the first treatment for MPSs: a one-year-old boy with MPS IH was given a bone-marrow transplant from his mother and presented some improvements (67). By far, over 1000 patients with various types of MPSs have been treated with HSCT, although therapeutic efficacy varies depending on the type of MPSs, age, clinical severity, and disease stage (68, 69). Variability of efficacy of HSCT for MPSs can be attributed to the fact that molecular environment of MPSs-related enzymes is too complicated. GT, a technique that modifies a person’s genes to treat or cure diseases, can work by several mechanisms: (1) Replacing a disease-causing gene with a healthy copy of the gene. (2) Introducing a new or modified gene into the body to help treat a disease. (3) Inactivating a disease-causing gene (70). GT is classified into in-vivo (vector is administered directly into the patient) and ex-vivo (vector is administered in cultured cells taken from the patient that are subsequently transplanted back) (71).

With careful selection and management, HSCT is a cost-effective therapeutic option for some groups of MPS patients (72). However, ex-vivo GT would be more stable than conventional HSCT. Firstly, ex-vivo GT is based on autologous HSCT to correct MPSs while conventional HSCT uses allogeneic cells, so ex-vivo GT can have lower risks of immune-mediated complications (69). Secondly, some elements in molecular environment can affect efficacy of HSCT through management of gene expression. Most of ex-vivo GTs replace original promoter by specific promoters that may be not affected by regulatory elements in molecular environment, so efficacy of GT may be more manageable (73). For example, ex-vivo GT with lentiviral vector expressing SGSH under the control of the CD11b myeloid-specific promoter presented compelling evidence of neurological disease correction in MPSIIIA mice (74). Based on pre-clinical data, a phase I/II clinical trial of ex-vivo GT for MPS IIIA was carried out (75). There is a risk that cell collection, culture, modification, and transplantation in ex-vivo GT lead to practical hurdles and face complicated molecular environment. Compared with ex-vivo GT, molecular environment of in-vivo GT is more controllable, because vector with functional gene is administered directly into the patient.

Gene editing has the ability to make highly specific changes in the DNA sequence of a living organism (76). Gene editing directly edits genome of patients with genetic disorder (genome editing) to rebuild molecular environment. Genome editing for MPSs has been reviewed by Poletto et al. (2020), who emphasized characteristics of genome editing (precise, definitive, and sometimes curative) (77). Although efficacy of genome editing remains to be proven, preliminary results of clinical trials are highly encouraging (78).

All approved therapies are ERTs, which demonstrates that ERT is the right way to treat MPSs patients (5). ERT really presents encouraging outcomes in reducing urinary GAGs and volume of liver and spleen, but effectiveness of ERT for cardiac valves, trachea and bronchi, central nervous system, hearing and eyes is definitely poor (79). Effectiveness of ERT for heart and joints are variable in different studies (79). Immunogenic responses and low-penetration in specific tissues may explain poor or variable effectiveness of ERT, but molecular environment also can give a hint for improvement of effectiveness. For example, alteration of cysteine residue to c-alpha formylglycine and phosphorylation at C-6 of mannose residue can affect efficacy of ERT (40, 45).

Atypical functions of enzymes associated with MPSs may have more influence on ERT and GT than on HSCT. One of the potential risks of GT is prolonged expression that can cause side effects because of atypical functions of MPSs-related enzymes. Inadequate cellular delivery in ERT causes uneven distribution and excess accumulation that may induce side effects too. Based on knowledge of molecular environment and atypical functions of MPSs-related enzymes, there will be more clear goals to improve efficacy and safety of therapies. Fusion with IGF2 to reduce unwanted delivery by enhancing lysosomal targeting and disrupting glycan structures is a good example (49, 51).

In summary, molecular environment and atypical functions of MPSs-related enzymes can affect efficacy and safety of therapies for patients with MPSs. To get more predictable results, specific modifications of MPSs-related enzymes were operated at the protein and gene level. However, the knowledge of molecular environment and atypical functions of MPSs-related enzymes is not enough for the specific modifications, so more attention should be paid to molecular environment and atypical functions of MPSs-related enzymes.

## Data Availability

Data from patients can be made available from the corresponding author after discussion with the Institutional Review Board.
